# Intestinal Immune Development Is Accompanied by Temporal Deviation in Microbiota Composition of Newly Hatched Pigeon Squabs

**DOI:** 10.1128/spectrum.01892-21

**Published:** 2022-05-17

**Authors:** Qianqian Xu, Wenyan Zhao, Yan Li, Xiaoting Zou, Xinyang Dong

**Affiliations:** a Key Laboratory for Molecular Animal Nutrition of the Ministry of Education, College of Animal Sciences, Zhejiang University, Hangzhou, China; Shandong First Medical University

**Keywords:** pigeon squabs, mucosal immune system, microbiota colonization, transcriptome, development

## Abstract

Identifying the interaction between intestinal mucosal immune system development and commensal microbiota colonization in neonates is of paramount importance for understanding how early life events affect resistance to disease later in life. However, knowledge about this interaction during the early posthatch development period in altrices is limited. To fill this gap, samples of intestinal content and tissue were collected from newly hatched pigeon squabs at four time points (days 0, 7, 14, and 21) for microbial community analysis and genome-wide transcriptome profiling, respectively. We show that the first week after hatching seems to be the critical window for ileal microbiota colonization and that a potentially stable microbiota has not yet been well established at 21 days of age. Regional transcriptome differences revealed that the jejunum rather than the ileum plays a crucial role in immunity at both the innate and adaptive levels. In the ileum, temporal deviation in innate immune-related genes mainly occurs in the first week of life and is accompanied by a temporal change in microbiota composition, indicating that the ileal innate mucosal immune system development regulated by microbial colonization occurs mainly in this period. Furthermore, we provide evidence that colonization by Escherichia and Lactobacillus within the first week of life is likely one of the causative factors for the induction of proinflammatory cytokine expression in the ileum. We also demonstrate that cellular adaptive immune responses mediated by Th17 cells following commensal-induced proinflammatory cytokine production in the ileum begin as early as the first week posthatch, but this cellular immunity seems to be less effective in terms of maintaining the inflammatory response balance. Because the induction of high levels of mucosal secretory IgA (SIgA) seems to take approximately 3 weeks, we favor the idea that humoral adaptive immunity might be less active, at least, during the first 2 weeks of life. Our data may help to explain the phenomenon of the occurrence of intestinal infections mainly in the ileum of pigeon squabs during the early posthatch period.

**IMPORTANCE** The pigeon (Columba livia), an altricial bird, is one of the most economically important farmed poultry for table purposes. Identifying the interaction between intestinal mucosal immune system development and commensal microbiota colonization in neonates is of paramount importance for understanding how early life events affect resistance to disease and potential productivity later in life. However, knowledge about this interaction during the early posthatch development period in altricial birds is limited. The study described herein is the first to try to provide insights into this interaction. Our data provide evidence on the mutual relationship between intestinal mucosal immune system development and commensal microbiota colonization in pigeon squabs and may help to explain the phenomenon of the occurrence of intestinal infections mainly in the ileum of pigeon squabs during the early posthatch period.

## INTRODUCTION

The small intestine plays a central part in the study of ontogeny in birds because of its role in digestion and nutrient absorption from the time of birth. However, knowledge about the normal development and functional maturation of the avian small intestine in the early posthatch period is scanty, except for studies on primarily domestic birds, such as chickens, that display a precocial developmental pattern (1–3). Birds that hatch ready to leave the nest and immediately proceed to forage an adult-type diet are called precocial, whereas birds that are unable to leave the nest and are fed processed foods or milk by parents are called altricial (4). The feeding habits of animals are reflected by the structure and function of the small intestine (2). As such, it is reasonable to predict that the pattern of small intestine development, both anatomically and functionally, would be different between precocial and altricial birds. The pigeon (Columba livia), an altricial bird, is one of China’s economically important farmed poultry and is widely distributed in southeastern areas of China. Squabs are a special kind of good for pigeon production. In 2018, squab production reached 250,000 tons in China. Knowledge about the intestinal development of pigeon squabs is still scarce and may be of paramount importance for understanding the effects of early life events on potential productivity later in life.

The microbiota and dietary antigens can trigger the development and functional maturation of the small intestine from the time of birth in mammals or after hatching in Aves. Studies in humans and laboratory animals have suggested a potential role for early-life microbial colonization in nutrient utilization, immune programming, and regulation of host physiology (5–7). Due to the resilience and stability of microbial communities (8), elucidating the properties of early-life microbial colonization may be useful to understanding the development of the entire small intestine in neonates. In Aves, although a few recent studies show that microbiota colonization begins in the embryonic stage (9, 10), in the days after hatching, the microbiota must ultimately develop from a simple, unstable community into a complex, established community. It has been shown that a potentially stable microbiota has not yet been well established at 14 days of age in precocial birds (11). Nevertheless, despite the clear importance of intestinal microbiota, our knowledge about the colonization and development of this complex ecosystem in altricial birds is still very poor.

Neonates are vulnerable to a variety of bacterial intestinal infections, not only because of an unstable microbiota community but also because of an immature immune system (12–14). The intestinal mucosal immune system development may involve maturation at both the innate and adaptive levels. Innate immunity includes a mechanical barrier formed by the intestinal epithelium, a mucus layer along the epithelial surface, complements, antimicrobial peptides, and cytokines secreted by innate immune cells (15). Tight junction (TJ) proteins, including claudins (CLDNs), occludins (OCLNs) and zonula occludens (ZOs), form the primary mechanical barrier and determine the permeability of the intestinal tract (15). Alterations in TJ proteins allow the translocation of luminal antigens (microorganisms and toxins) into the whole body through the mucosa, where these antigens are usually excluded and subsequently disrupt mucosal homeostasis (16). The secreted mucins are key components of the protective mucus layer, acting from lubrication to cell signal transduction to the formation of a barrier against pathogens and chemicals (17). The complement system is composed of a tightly regulated network of proteins that play a pivotal role in the maintenance of host defense (18). Antimicrobial peptides participate in host defense and inflammation due to their broad-spectrum antimicrobial activity (19). Cytokines secreted by innate immune cells, such as tumor necrosis factor alpha (TNF-α) and interleukin-1 beta (IL-1B), can control the microbiota by activating and polarizing T helper (Th) cells (20). Adaptive immunity consists of two main components, T and B cells. Naive CD4^+^ T cells can be classified into Th subsets (Th1, Th2, and Th17) and regulatory T cells (Treg), and these subsets antagonize or restrict each other by generating their effector cytokines (21). It is well known that the Th1/Th2/Th17/Treg balance, also known as the proinflammatory/anti-inflammatory cytokine balance, plays a major role in maintaining immune homeostasis in animals and humans (22). Secretory IgA (SIgA), generated from effector B cells, is required to mediate the humoral adaptive immune response at mucosal surfaces (23). In addition, studies in mammals and birds have suggested that SIgA contributes to the maintenance of mucosal homeostasis due to its role in the downmodulation of bacterially mediated inflammation (24, 25). The above-mentioned descriptions imply that the colonization of the microbiota and the development of the immune system at both the innate and adaptive levels are intertwined with each other. The dialogue between the intestinal mucosal immune cells and the microbiome is mainly through the pattern recognition receptors (PRRs), such as NOD-like receptors (NLRs) and Toll-like receptors (TLRs).

A grand challenge for developing the intestinal mucosal immune system is to distinguish between pathogenic microorganisms and commensal microorganisms without interfering with the intestinal functions necessary to maintain host health (26). Evidence in precocial chicks has shown that functional maturation of the innate and adaptive immune systems occurs mainly in the first 2 weeks after hatching (3, 27), and this maturation occurs along with the temporal changes in the microbiota composition (20). In addition, the role of the intestinal microbiome in mucosal immune system development through its cross talk with epithelial and immune cells is a well-established area in the microbiology of vertebrate hosts (15). However, knowledge about this role of microbiota in altricial birds is limited. The study described herein is the first attempt to provide insights into interactions between intestinal mucosal immune system development and commensal microbiota colonization in an altricial bird and may help in understanding how the early life events affect resistance to disease later in life. As such, in the current study, we initially investigated the microbial community profile of the small intestine in pigeon squabs during the early posthatch period. Then, genome-wide transcriptome profiling (RNA-Seq) of intestinal mucosal tissue was also carried out, which was mainly focused on the intestinal mucosal immune system-related genes, and the results were subsequently related to the microbial community profiles.

## RESULTS

### Microbiota analyses.

**FIG 1:**
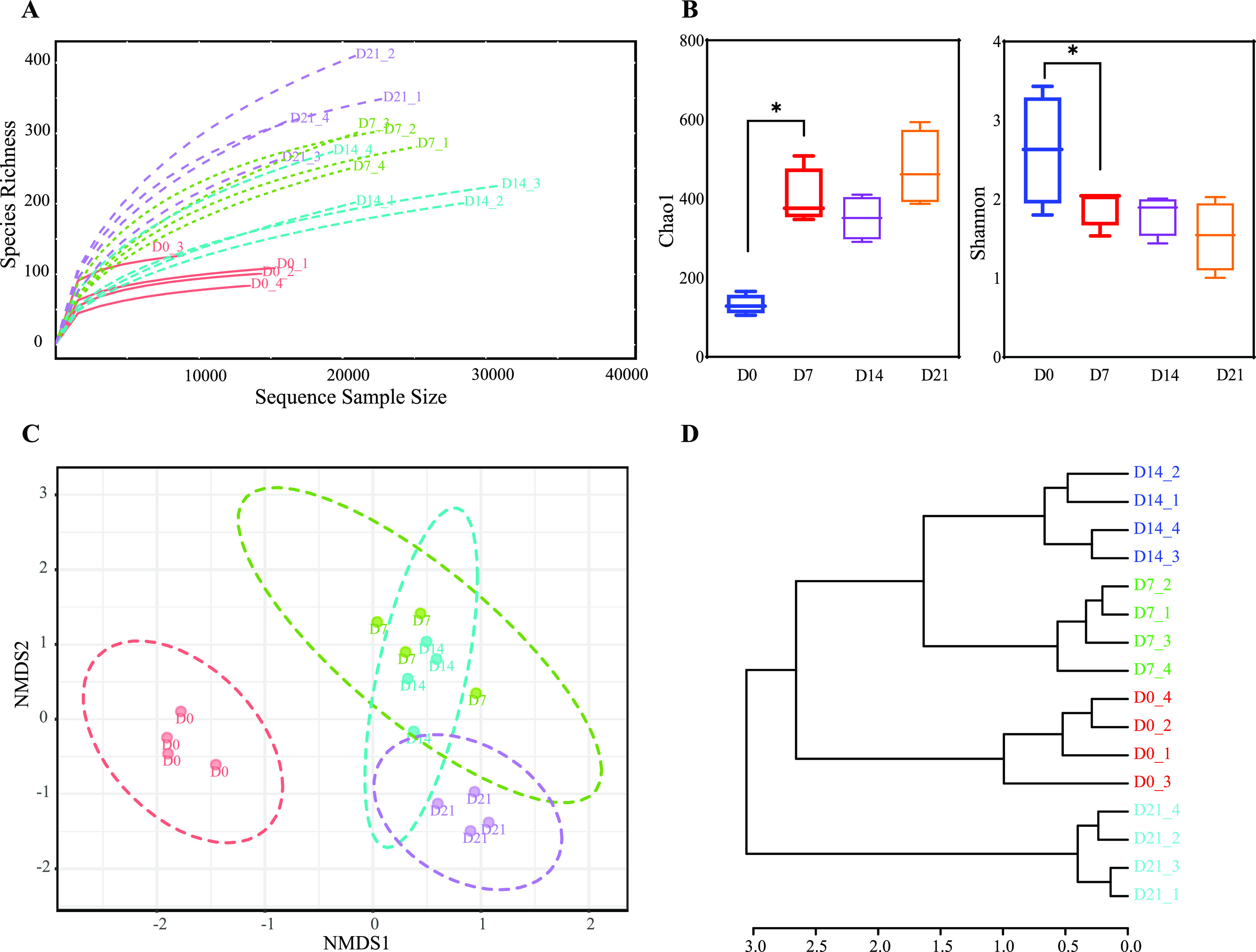
Differences in bacterial community diversity, richness, and structure between pigeon squabs for four age groups. (A) Rarefaction curve. (B) Box plot of the microbiota diversity and richness represented by Chao1 and the Shannon index. *, significantly different by Kruskal-Wallis test (*P* < 0.05). Error bars show standard errors. (C) NMDS plot of bacterial communities with 80% confidence ellipses according to the Bray-Curtis phylogenetic distance metric. Each symbol represents the data from pooled samples from 3 squabs. (D) Hierarchical clustering of the microarray data according to the Bray-Curtis phylogenetic distance metric.

An average of 21,315 high-quality sequences per sample were obtained from 16 ileal content samples from pigeon squabs at day zero (D0), D7, D14, and D21. Furthermore, an average of 259 (ranging from 99 to 448) operational taxonomic units (OTUs) were identified from these sequences according to 97% sequence similarity (Data Set S2 in the supplemental material). As the number of sequences increased, the rarefaction curve of each sample leveled off (Fig. 1A), suggesting that the number of sequences generated in the current study covered most of the existing bacterial diversity within the pigeon squab’s intestinal microbiota. Chao1 increased and the Shannon index decreased significantly during the first week after hatching, but no further change was detected after D7 (Fig. 1B). Nonmetric multidimensional scaling (NMDS), a multivariate statistical method, was applied to examine correlations between the microbes at different time points. The NMDS ordination, as shown in Fig. 1C, based on permutational multivariate analysis of variance (MANOVA) of Bray-Curtis distance metrics, showed that samples were clearly clustered according to development stage in this study. In addition, birds aged 7 to 21 days clustered together in the right part of the graph, whereas birds of D0 mainly clustered in the left part. This dissimilarity of the microbiota compositions was also confirmed by the separately clustered microbiota of all age groups shown in hierarchical clustering plots (Fig. 1D).

**FIG 2:**
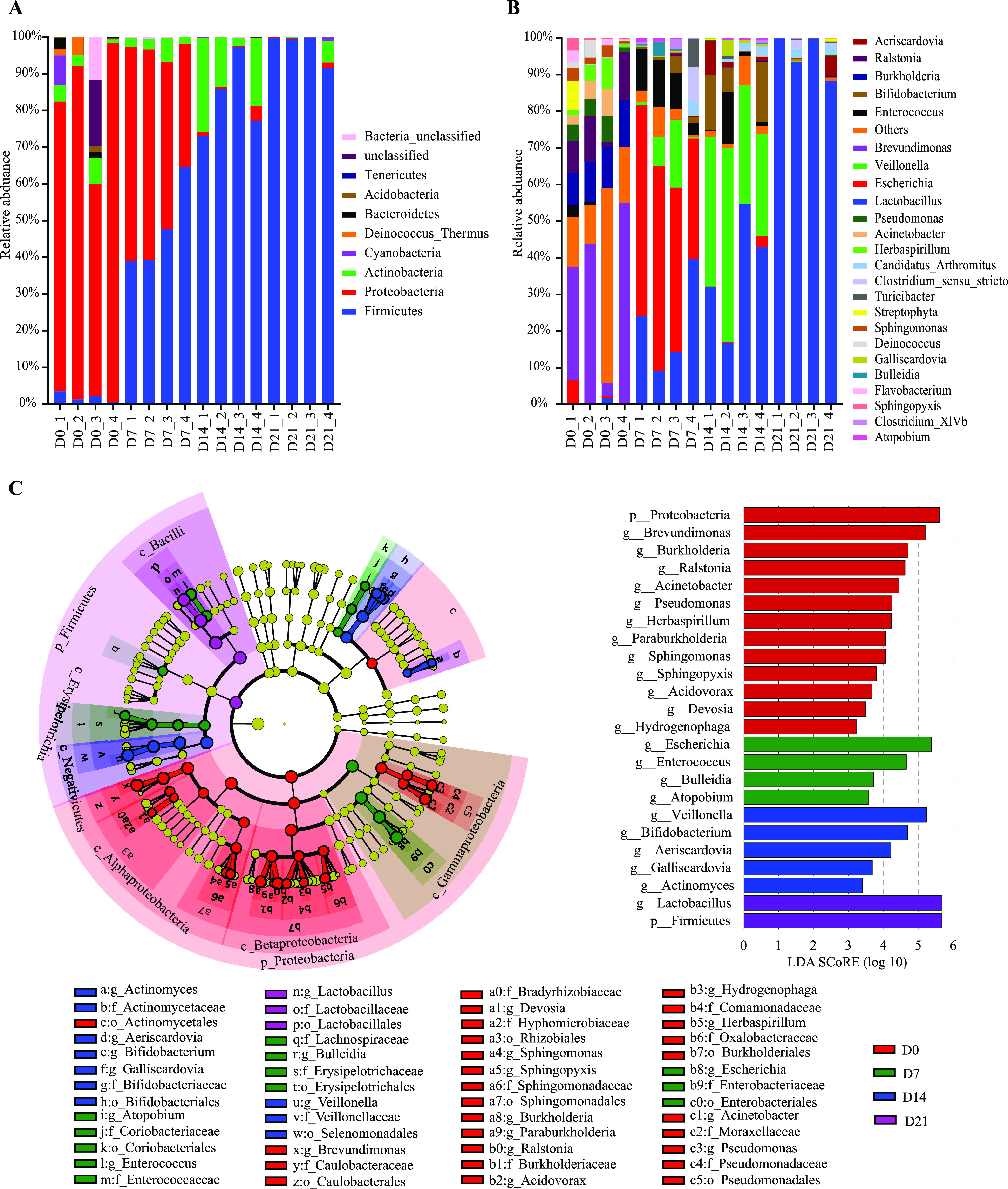
Temporal variations of microbial compositions in the pigeon squabs. (A) Relative abundances of microbial communities at the phylum level. (B) Relative abundances of microbial communities at the genus level. (C) Cladogram of enriched taxa obtained from LEfSe analysis shows significant differences (Kruskal-Wallis test, *P* < 0.05) in the microbial communities between groups. p, phylum; o, order; f, family; g, genus.

The relative abundances of microbiota compositions with respect to the levels of phylum and genus (the 25 most abundant genera across all age groups) are shown in Fig. 2A and B and Data Set S3. The microbial communities changed significantly with age at both the phylum and genus levels. Immediately after hatching, the microbiota was characterized by a high abundance of Proteobacteria, which formed more than 80% of the total microbial population. Pigeon squabs at D21 were characterized by a drop in Proteobacteria to less than 1% and by nearly absolute dominance of the representatives of Firmicutes, which formed approximately 97% of the microbiota. At the genus level, Proteobacteria were represented mainly by Brevundimonas, Burkholderia, and Ralstonia in pigeon squabs at D0. Interestingly, Escherichia, belonging to the phylum Proteobacteria, was common and predominant in all samples at D7, ranging from 33% to 58% relative abundance. However, Escherichia was outcompeted by representatives of Firmicutes (mainly of the genus Veillonella and Lactobacillus) and Actinobacteria (mainly of the genus Bifidobacterium) at D14. At D21, Lactobacillus became the predominant genus in pigeon squabs, exceeding 90% relative abundance.

Significant changes in microbiota compositions over time were also observed by linear discriminant analysis effect size (LEfSe) analysis (Fig. 2C). At the genus level, the relative abundances of Brevundimonas, Burkholderia, Ralstonia, Acinetobacter, Herbaspirillum, Pseudomonas, Paraburkholderia, Sphingomonas, Sphingopyxis, Acidovorax, Devosia, and Hydrogenophaga showed remarkable decreases when comparing D7 to D0 but showed no further change after D7. In contrast, the relative abundance of Lactobacillus increased as the birds aged and displayed the highest level at D21. Escherichia, Enterococcus, Bulleidia, and Atopobium showed increases between D0 and D7 but subsequently decreased with age. Veillonella, Bifidobacterium, Aeriscardovia, Galliscardovia, and Actinomyces showed the highest abundances at D14.

### Transcriptome analyses.

**FIG 3:**
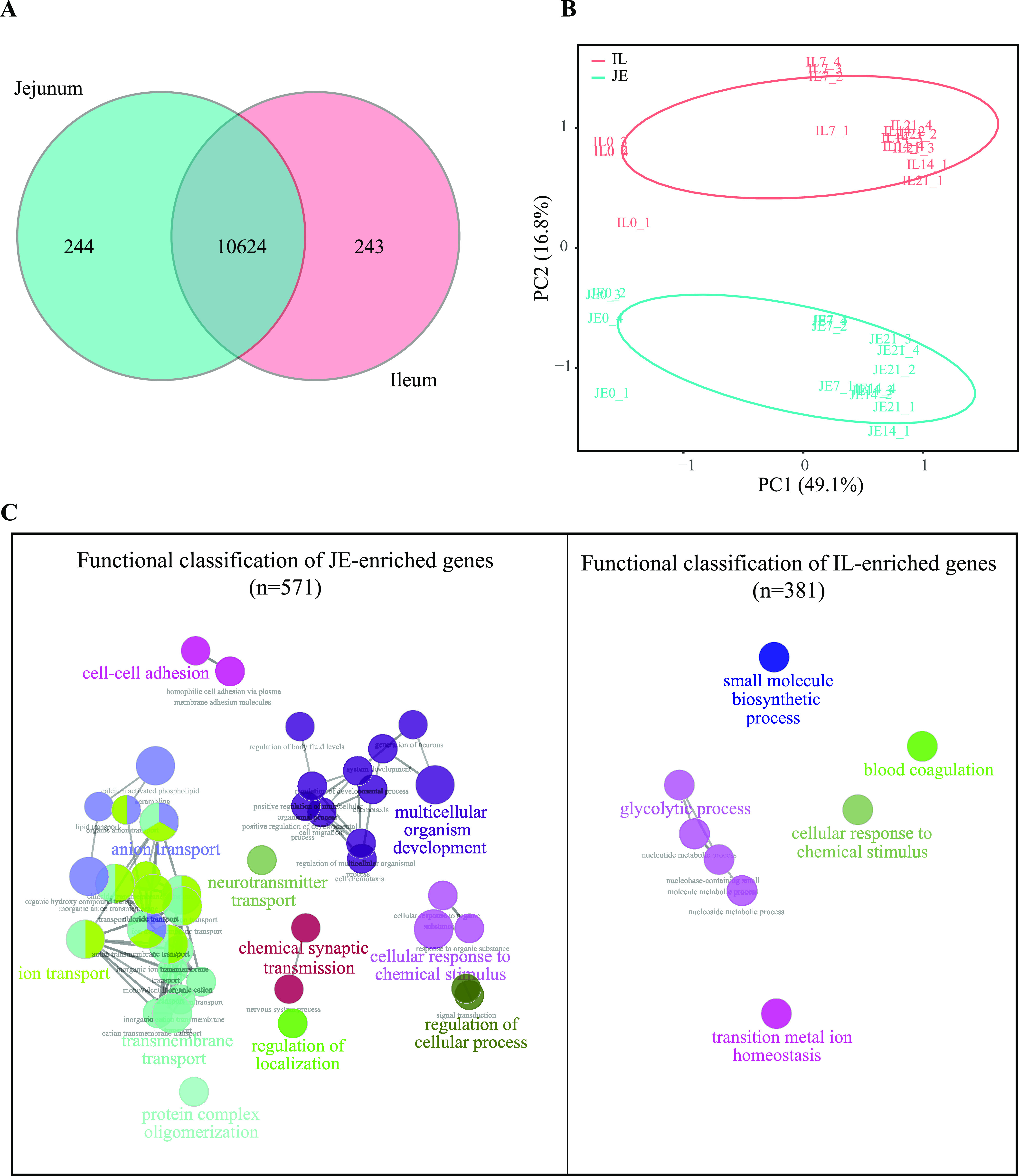
Differences between the genes expressed in the jejunum (JE) and ileum (IL) from transcriptome analysis. (A) Venn diagram of all the expressed genes. (B) PCA plot of transcriptome profile data. Each symbol represents the data from pooled samples from 3 squabs. (C) Clusters of enriched GO terms obtained from regionally DE genes. Each node depicts a GO Term. Different node colors represent different functional groups based on the kappa value. The most significant GO terms are highlighted in color. The node size is proportional to the significance of the GO term. Functionally associated groups partially overlap.

In total, 846 million high-quality clean reads were generated from 32 cDNA libraries (samples of jejunal and ileal tissue from pigeon squabs at D0, D7, D14, and D21), with an average of 26.44 ± 2.88 million (mean ± standard deviation) clean reads from each library. Of all the high-quality clean reads, an average of 78.24% from the ileum and 76.95% from the jejunum were uniquely mapped to the Columba livia reference genome. In addition, the genic distribution of all the uniquely mapped reads showed that 86.22% of them mapped to protein coding genes, 5.36% of them were distributed among introns, and 8.42% of them were distributed among intergenic regions. A total of 11,111 expressed protein coding genes were detected based on the normalized data (Data Set S4). Among these expressed protein coding genes, 10,624 genes were commonly detected in both the ileum and jejunum, as shown in Fig. 3A. Explorative principal-component analysis (PCA) was performed, in which the clustering pattern of the transcriptome profiles generated in pigeon squabs was clearly dependent on the intestinal region rather than the age of the birds (Fig. 3B). The regional differences across all age groups were further analyzed using the DESeq R package. We found that 571 genes were expressed at higher levels in the jejunum (Fig. 3C, JE-enriched genes) than in the ileum at one or more time points, while 381 genes were expressed at higher levels in the ileum (Fig. 3C, IL-enriched genes). In this study, ClueGO-mediated enrichment analysis was conducted to annotate the biological functions of these regionally differentially expressed (DE) genes. Forty-two significantly enriched GO terms in the jejunum could be classified into 11 related functional groups, while 8 significantly enriched GO terms in the ileum were classified into 5 related functional groups (Fig. 3C and Data Set S5). The results revealed that JE-enriched genes were primarily correlated with multicellular organism development and transport, while IL-enriched genes were primarily correlated with the glycolytic process.

**FIG 4:**
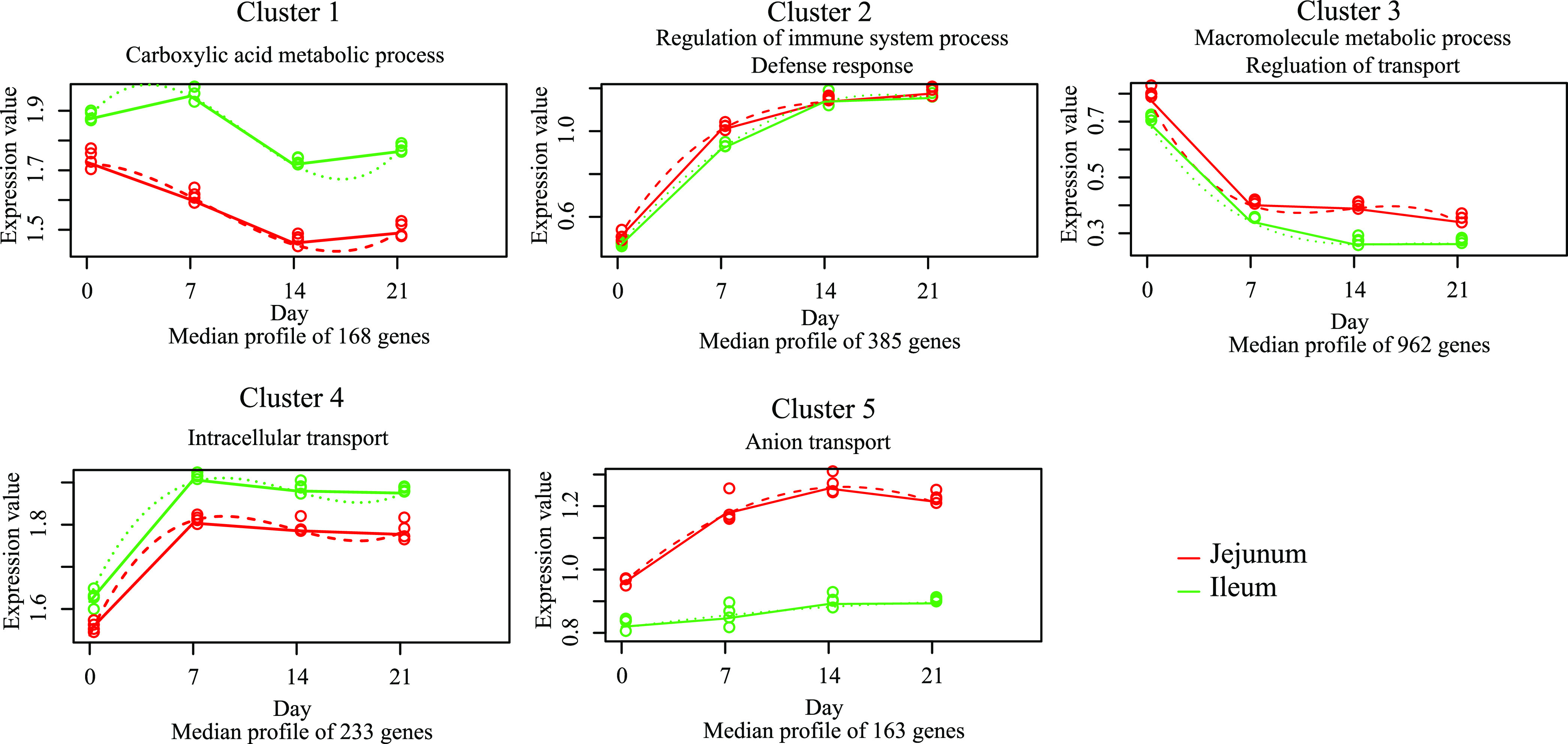
Cluster analysis of DE genes during pigeon squab development. Each cluster consists of multiple genes displaying differential temporal gene expression patterns between jejunum and ileum. *x* axis shows the development stages. *y* axis shows the gene expression values [log_10_(FPKM + 1)]. The gene expression values are represented by solid lines, while the predictions from the regression-based analysis are represented by dotted lines. Functional annotation clustering was conducted to explore the biological functions of the DE genes in a certain cluster, and representative GO terms for each cluster are represented above the graph.

We used a regression-based method to explore the differences in temporal gene expression patterns between the jejunum and ileum. A total of 1,911 temporally DE genes were associated with development stage. The expression patterns of these temporally DE genes were grouped into 5 clusters (Fig. 4). Most of the temporally DE genes showed a change in expression immediately after hatching in both the jejunum and ileum. From all the patterns observed, two patterns (clusters 2 and 3) represented 70% of the genes for both tissues. Genes in cluster 2 displayed a quadratic increase in expression during the first 3 weeks, whereas genes in cluster 3 displayed a quadratic decrease. In addition, functional annotation clustering was conducted to explore the biological functions of the temporally DE genes in a certain cluster, and the results are shown in Data Set S6, with representative GO terms depicted in Fig. 4. For instance, temporally DE genes within cluster 2 were mainly related to immune functions, and temporally DE genes within cluster 3 were mainly related to metabolic and transport functions.

**FIG 5:**
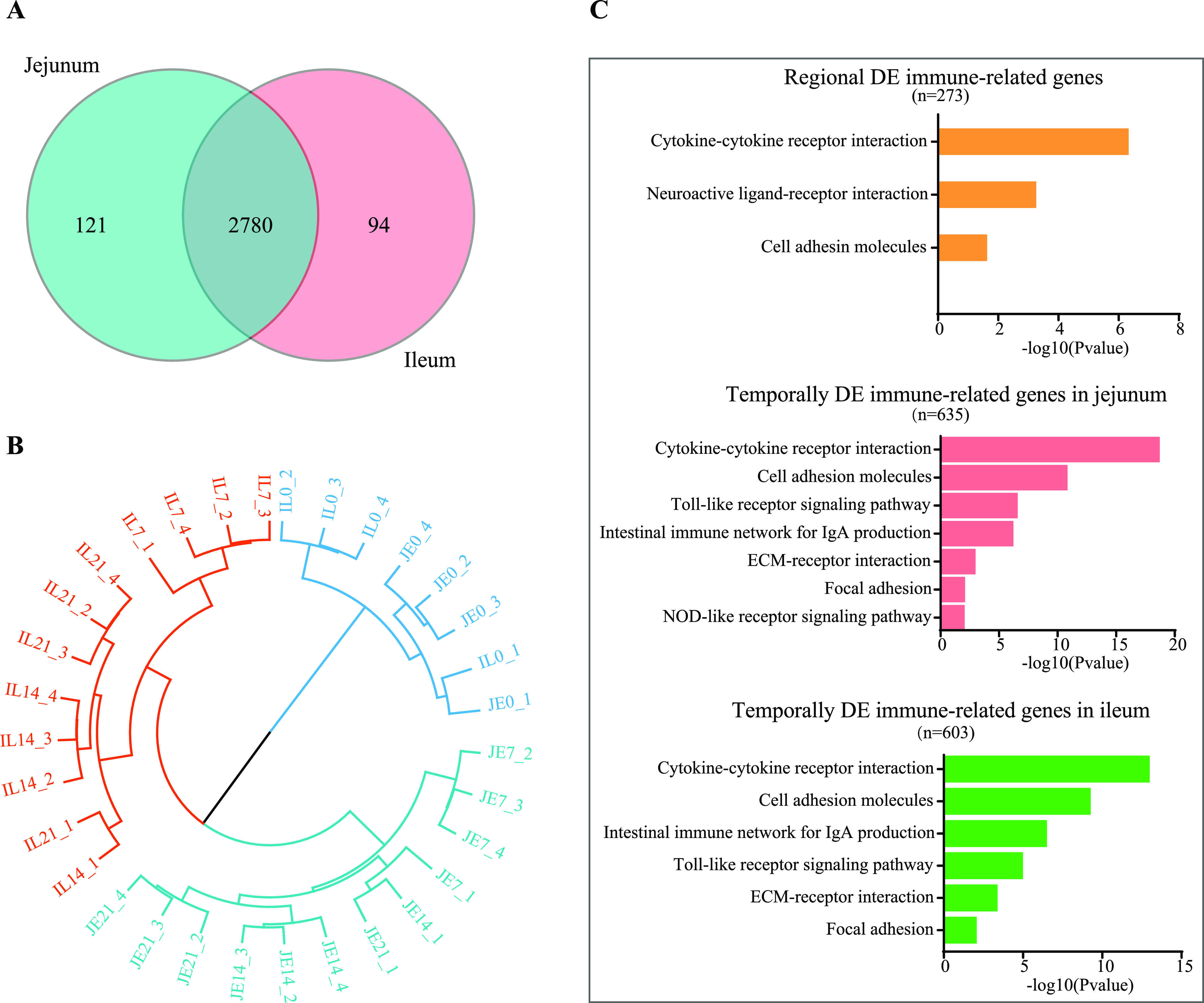
Overview of regional and temporal differences in immune-related genes. (A) Venn diagram of all the expressed immune-related genes. (B) Hierarchical clustering of gene expression. Each symbol represents the data from pooled samples from 3 squabs. JE, jejunum; IL, ileum. (C) KEGG pathway analysis of regionally and temporally DE immune-related genes.

To obtain an overview of regional and temporal differences in the development of the intestinal mucosal immune system in pigeon squabs, a total of 2,995 expressed immune-related genes (Fig. 5A and Data Set S7) were subjected to analysis. A clear separation of the samples was observed based on the hierarchical clustering analysis, in which the samples were segregated by the ages of the birds (D0 versus D7, D14, and D21), then by intestinal region (jejunum versus ileum), and finally by age again (D14 versus D7 and D21 versus D14) (Fig. 5B). When the expressed immune-related genes were compared between the jejunum and ileum, 273 regionally DE genes were identified (Data Set S8). These regionally DE immune-related genes were related to cytokine-cytokine receptor interaction, neuroactive ligand-receptor interaction, and cell adhesin molecules, based on the Kyoto Encyclopedia of Genes and Genomes (KEGG) pathway analysis (Fig. 5C). Temporal expression pattern analysis of the expressed immune-related genes revealed that 635 were DE in the jejunum and 603 were DE in the ileum during the three posthatch periods (Data Set S9). KEGG pathway analysis showed that 7 and 6 pathways were significantly altered in the jejunum and ileum, respectively, 6 of which overlapped in both segments.

### Expression patterns of innate immune-related genes.

**FIG 6:**
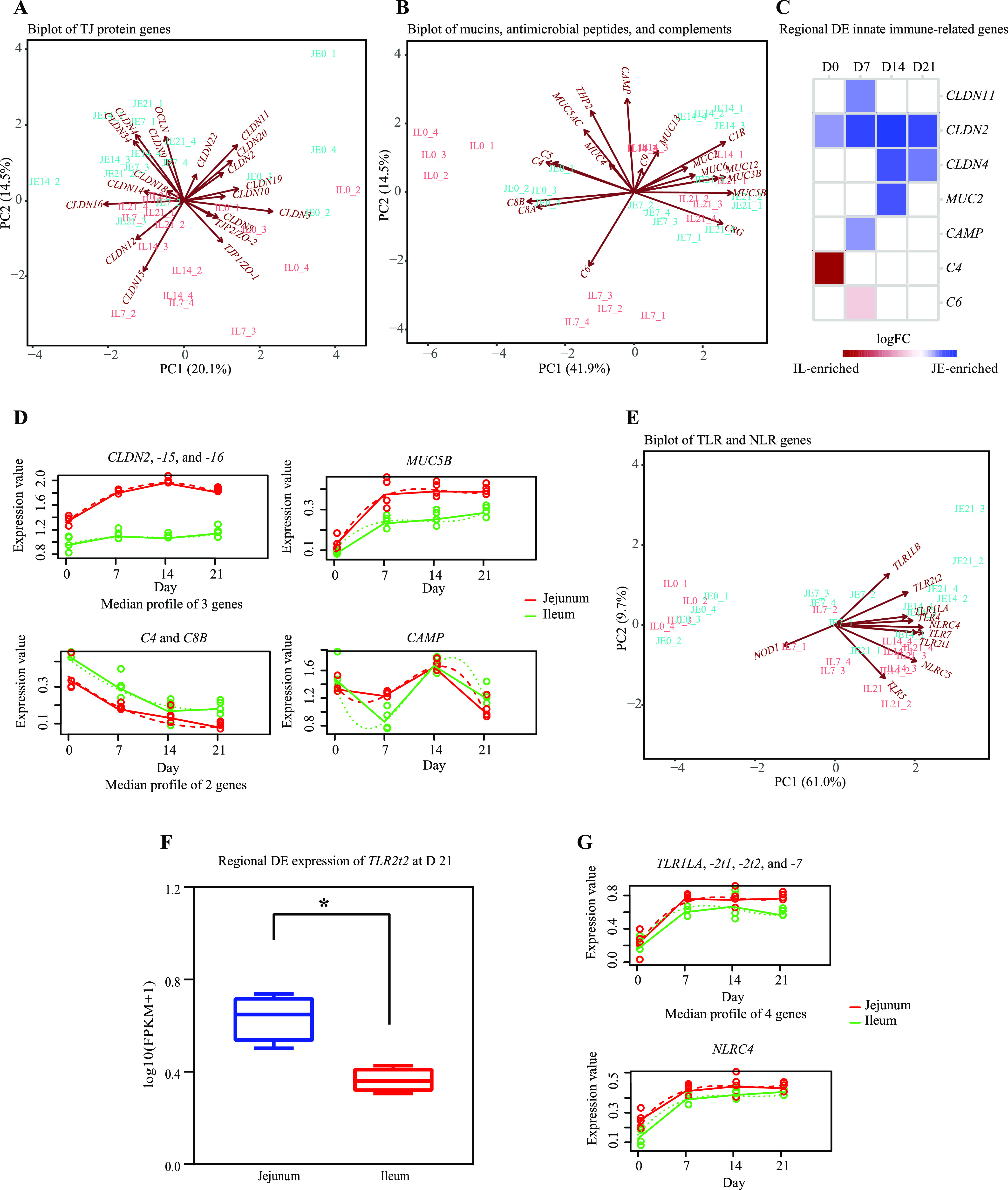
The expression pattern of innate immune-related genes in the jejunum (JE) and ileum (IL). (A) Biplot of TJ protein genes. Each symbol represents the data from pooled samples from 3 squabs. (B) Biplot of mucin, antimicrobial peptide, and complement genes. Each symbol represents the data from pooled samples from 3 squabs. (C) Regional differential expression analysis of innate immune-related genes. Blue indicates high expression in jejunum, and red indicates high expression in ileum. (D) Temporal differential expression analysis of innate immune-related genes. *x* axis shows the development stages. *y* axis shows the gene expression values [log_10_(FPKM + 1)]. The gene expression values are represented by solid lines, while the predictions from the regression-based analysis are represented by dotted lines. (E) Biplot of TLR and NLR genes. Each symbol represents the data from pooled samples from 3 squabs. (F) The expression pattern of *TLR2t2* in the small intestine. *, significant difference obtained from regional differential expression analysis. Error bars show standard errors. (G) Temporal differential expression analysis of TLR and NLR genes. *x* axis shows the development stages. *y* axis shows the gene expression values [log_10_(FPKM + 1)]. The gene expression values are represented by solid lines, while the predictions from the regression-based analysis are represented by dotted lines.

The expression of innate immune-related genes, including genes encoding TJ proteins (*CLDN2*, *-3*, *-4*, *-8*, *-9*, *-10*, *-11*, *-12*, *-14*, *-15*, *-16*, *-18*, *-19*, *-20*, *-22*, and *-34*, *OCLN*, *ZO-1*, and *ZO-2*), mucins (*MUC2*, *-3B*, *-4*, *-5AC*, *-5B*, *6*, *-12*, and *-13*), antimicrobial peptides (cathelicidin antimicrobial peptide [*CAMP*] and beta-defensin 2 [*THP2*]), and complements (*C1R*, *-4*, *-5*, *-6*, *-8A*, *-8B*, *-8G*, and -*9*), was observed in the jejunal and ileal tissue samples of pigeon squabs (Fig. 6A and B). Between the two intestinal regions, *CLDN2*, *CLDN4*, *CLDN11*, *MUC2*, and *CAMP* were expressed at higher levels in the jejunum than in the ileum mainly after D7, while *C4* and *C6* were IL-enriched genes during the first week of life (Fig. 6C). When comparing the expression of these genes across all age groups, the expression of *CLDN2*, *CLDN15*, and *CLDN16* showed remarkable increases on D7 compared to their expression on D0 in the jejunum but showed no significant changes among the four age groups in the ileum (Fig. 6D). *MUC5B* expression showed marked increases during the first week of life in both the jejunum and ileum but showed no further change after D7. *CAMP* expression decreased cubically in response to increased age, with the highest expression levels observed at D0 and D14 in either the jejunum or the ileum. Both *C4* and *C8B* showed decreased expression in the jejunum within the first 3 weeks posthatch and in the ileum within the first 2 weeks after hatching.

### Expression pattern of innate immune sensing-related genes.

The expression of TLR genes (*TLR1LA*, *TLR1LB*, *TLR2t1*, *TLR2t2*, *TLR4*, *TLR5*, and *TLR7*) and NLR genes (*NOD1*, *NLRC4*, and *NLRC5*) was observed in the jejunal and ileal tissue samples of pigeon squabs, and it segregated by age due to lower expression levels at D0 compared with the expression levels at older ages (Fig. 6E). Between the two intestinal regions, only *TLR2t2* was identified as regionally DE, with higher expression observed at D21 in the jejunum than in the ileum (Fig. 6F). All temporally DE TLR and NLR genes showed markedly increased expression in both the jejunum and ileum between D0 and D7 (Fig. 6G).

### Expression pattern of T- and B-cell lineage-specific genes.

**FIG 7:**
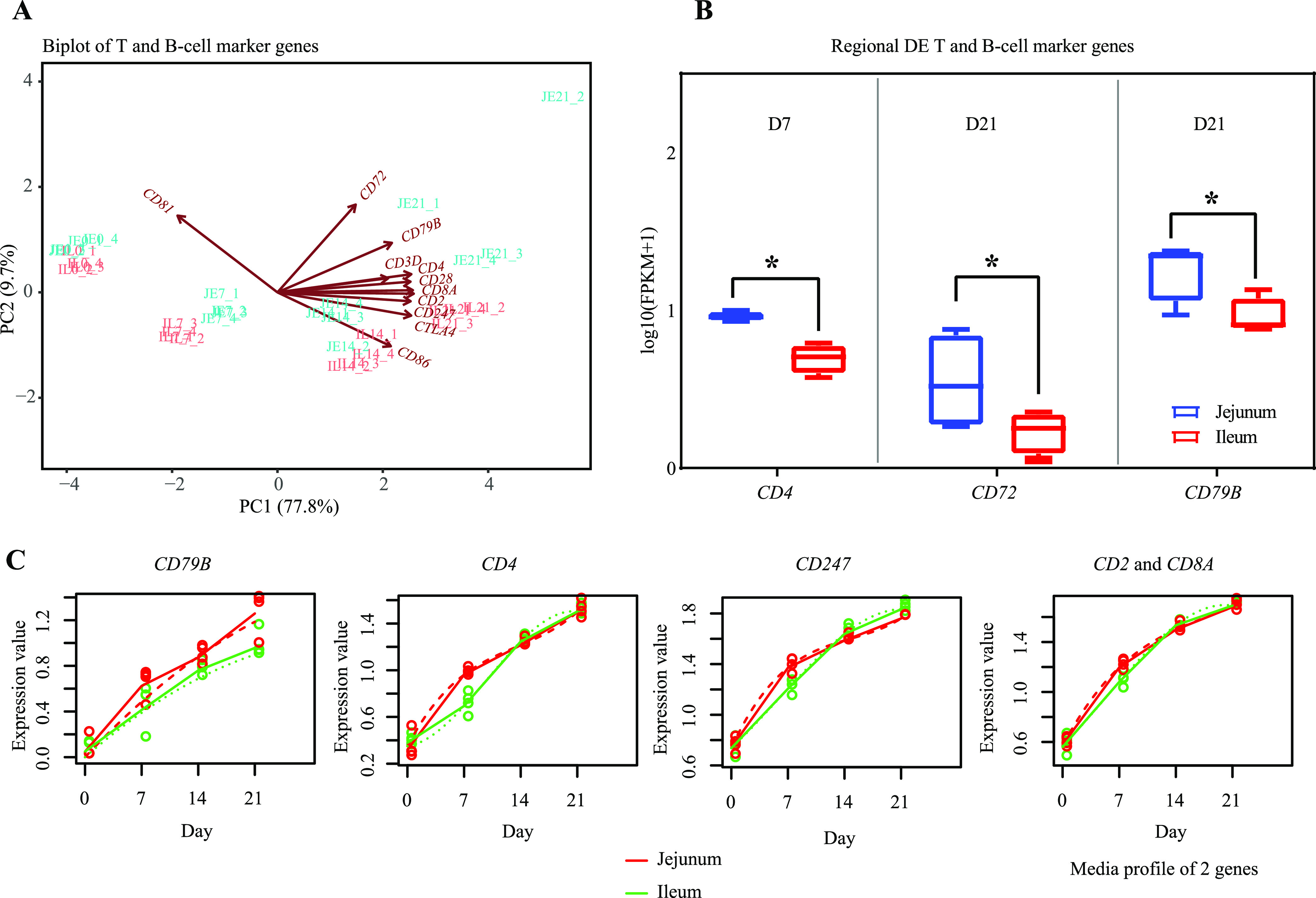
The expression pattern of T- and B-cell lineage-specific genes in the jejunum (JE) and ileum (IL). (A) Biplot of T- and B-cell marker genes. Each symbol represents the data from pooled samples from 3 squabs. (B) The expression pattern of T- and B-cell marker genes in the small intestine. *, significant difference obtained from regional differential expression analysis. Error bars show standard errors. (C) Temporal differential expression analysis of T- and B-cell marker genes. *x* axis shows the development stages. *y* axis shows the gene expression values [log_10_(FPKM + 1)]. The gene expression values are represented by solid lines, while the predictions from the regression-based analysis are represented by dotted lines.

The expression of T-cell lineage-specific genes (cluster of differentiation 2 [*CD2*], *-247*, *-28*, *-3D*, *-4*, and *-8A* and cytotoxic T-lymphocyte-associated protein 4 [*CTLA4*]) and B-cell lineage-specific genes (*CD79B*, *-86*, *-81*, and *-72*) was observed in the jejunal and ileal tissue samples of pigeon squabs and segregated by age due to lower expression levels at either D0 or D7 compared with older ages (Fig. 7A). Between the two intestinal regions, *CD4*, *CD72*, and *CD79B* were identified as regionally DE, with higher expression observed at either D7 or D21 in the jejunum than in the ileum (Fig. 7B). All temporally DE T- and B-cell marker genes showed markedly increased expression through the first 3 weeks after hatching in both the jejunum and ileum (Fig. 7C).

### Cytokine gene expression pattern and IgA production analysis.

**FIG 8:**
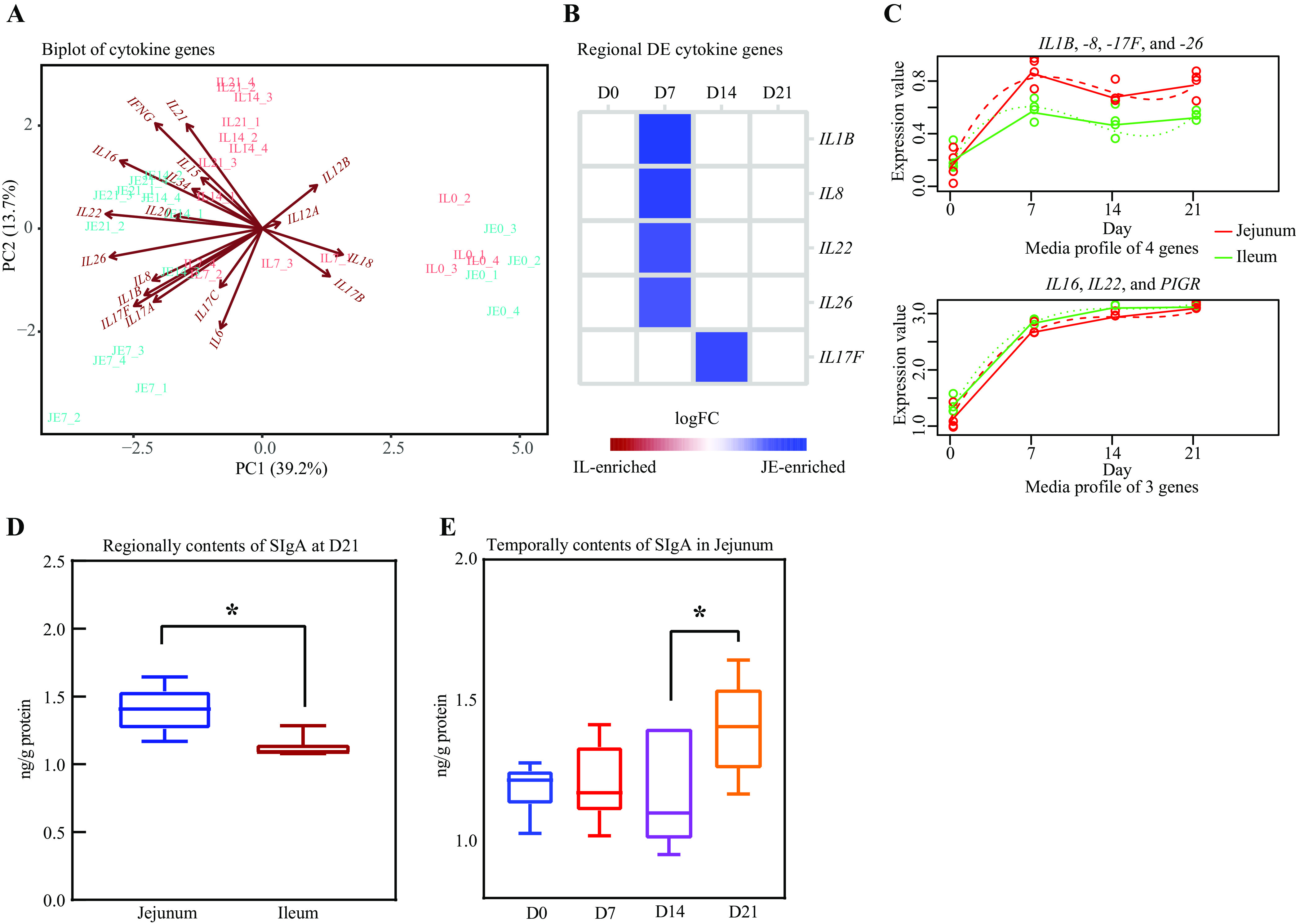
Cytokine gene expression pattern and IgA production analysis. (A) Biplot of cytokine genes. Each symbol represents the data from pooled samples from 3 squabs. (B) Regional differential expression analysis of cytokine genes. Blue indicates high expression in jejunum. (C) Temporal differential expression analysis of cytokine genes and polymeric immunoglobulin receptor gene (*PIGR*). *x* axis shows the development stages. *y* axis shows the gene expression values [log_10_(FPKM + 1)]. The gene expression values are represented by solid lines, while the predictions from the regression-based analysis are represented by dotted lines. (D) Regional contents of secretory IgA (SIgA) at D21. *, significant difference between jejunum and ileum (*P* < 0.05). *y* axis depicts the content levels. (E) Temporal contents of SIgA in the jejunum. *, significant difference between proximal age groups (*P* < 0.05). Error bars show standard errors.

Expression of cytokine genes (interleukin genes *IL12A*, *-12B*, *-16*, *-15*, *-17A*, *-17B*, *-17C*, *-17F*, *-18*, *-1B*, *-20*, *-21*, *-22*, *-26*, *-34*, *-6*, and *-8* and gamma interferon [IFN-γ] gene *IFNG*) was observed in the jejunal and ileal tissue samples of pigeon squabs (Fig. 8A). Between the two intestinal regions, *IL1B*, *IL8*, *IL2*, *IL26*, and *IL17F* were identified as regionally DE, with higher expression observed at or after D7 in the jejunum than in the ileum (Fig. 8B). Temporal analysis showed that the increase in interleukin expression occurred mainly during the first week of life (Fig. 8C). The polymeric immunoglobulin receptor gene (*PIGR*) was temporally DE, and its expression in both the jejunum and ileum showed an increase within the first week (Fig. 8C). In addition, the SIgA content was higher in the jejunal mucosa than in the ileal mucosa at D21 (Fig. 8D). When the SIgA content was compared for different ages in the jejunum, it was significantly higher at D21 than at D14 (Fig. 8E). No temporal effect was detected for the content of SIgA in the ileum (data not shown).

### Correlations of specific taxa with immune-related gene expression.

As can be seen by the results in Table 1, the temporal change of Atopobium was positively correlated with those of *TLR1LA* and *TLR2t2* at week 1 and positively correlated with that of *NLRC4* at week 3. The temporal change of Enterococcus was positively correlated with those of *TLR1LA*, *TLR2t2*, *TLR2t1*, *TLR7*, and *NLRC4* at week 1 while being negatively correlated with that of *TLR2t2* at week 2. The temporal change of Escherichia was positively correlated with those of *TLR1LA*, *TLR2t2*, *TLR2t1*, *TLR7*, *NLRC4*, *IL1B*, and *IL8* at week 1 while being negatively correlated with those of *TLR7*, *NLRC4*, and *IL8* at week 2. The temporal change of Lactobacillus was positively correlated with those of *TLR1LA*, *TLR2t2*, *TLR2t1*, *NLRC4*, *IL1B*, and *IL8* at week 1 and positively correlated with that of *TLR2t1* at week 2. The temporal change of Veillonella was positively correlated with those of *TLR7* and *NLRC4* at week 2.

**TABLE 1:** Correlations between bacterial abundance and intestinal gene expression in pigeon squabs

Age of squabs (wk)	Bacterial genus	Correlation of bacterial abundance and expression level of^*a*^:						
		*TLR1LA*	*TLR2t2*	*TLR2t1*	*TLR7*	*NLRC4*	*IL1B*	*IL8*
1	*Atopobium*	0.77*	0.73*	NS	NS	NS	NS	NS
	*Enterococcus*	0.82*	0.83*	0.71*	0.81*	0.76*	NS	NS
	Escherichia	0.94**	0.96**	0.88**	0.90**	0.90**	0.75*	0.73*
	*Lactobacillus*	0.76*	0.81*	0.83*	NS	0.81*	0.73*	0.85**
	*Bifidobacterium*	NS	NS	NS	NS	NS	NS	NS
	*Veillonella*	NS	NS	NS	NS	NS	NS	NS

2	*Atopobium*	NS	NS	NS	NS	NS	NS	NS
	*Enterococcus*	NS	NS	−0.80*	NS	NS	NS	NS
	Escherichia	NS	NS	NS	−0.91**	−0.90**	NS	−0.93**
	*Lactobacillus*	NS	NS	0.74*	NS	NS	NS	NS
	*Bifidobacterium*	NS	NS	NS	NS	NS	NS	NS
	*Veillonella*	NS	NS	NS	0.83*	0.72*	NS	NS

3	*Atopobium*	NS	NS	NS	NS	0.83*	NS	NS
	*Enterococcus*	NS	NS	NS	NS	NS	NS	NS
	Escherichia	NS	NS	NS	NS	NS	NS	NS
	*Lactobacillus*	NS	NS	NS	NS	NS	NS	NS
	*Bifidobacterium*	NS	NS	NS	NS	NS	NS	NS
	*Veillonella*	NS	NS	NS	NS	NS	NS	NS

a Significant correlation between two indices (two-tailed) is shown as follows: *, *P* < 0.05; **, *P* < 0.01; two-tailed. NS, not significant.

### Validation of DE mRNAs.

**FIG 9:**
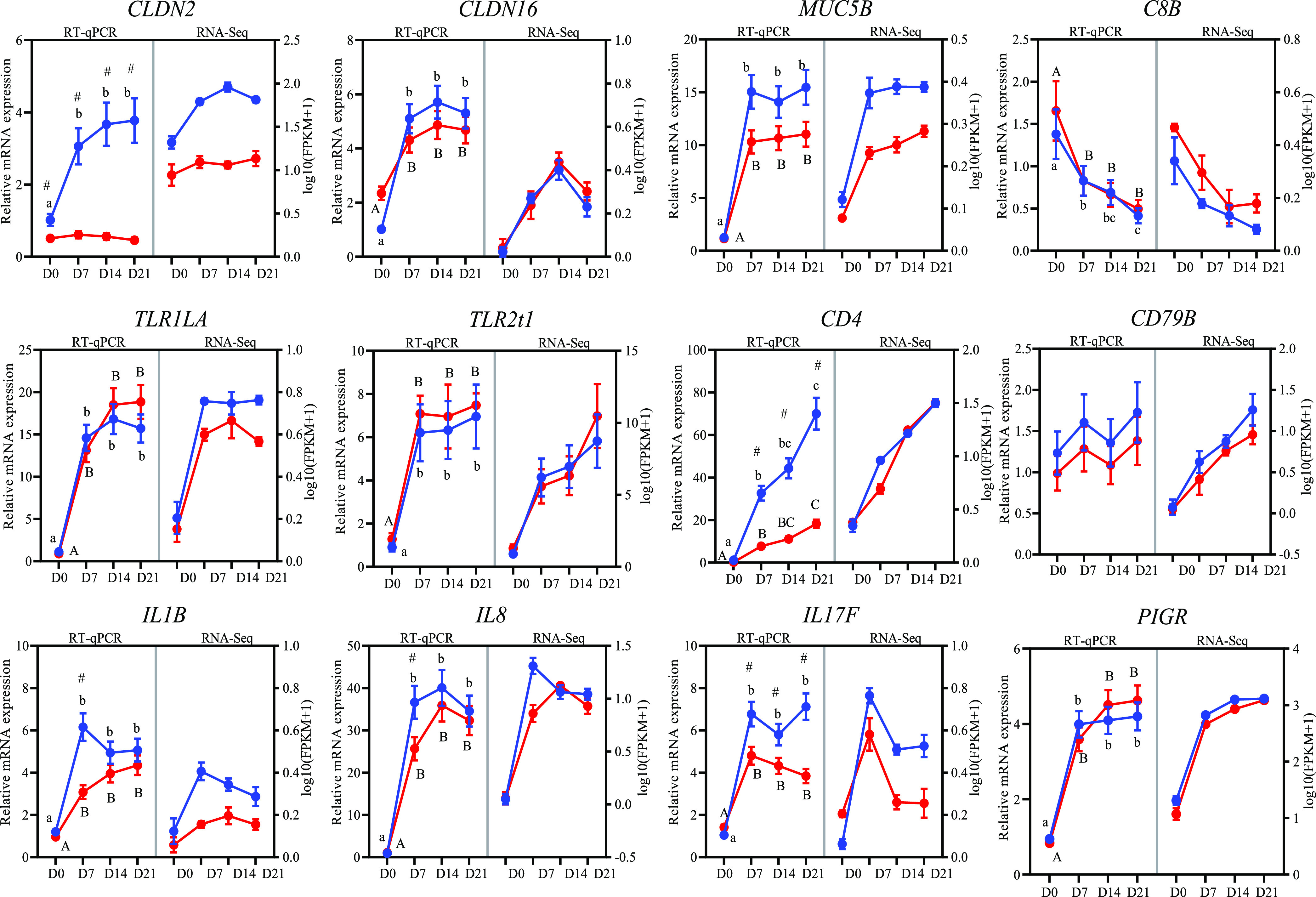
Validation of the selected DE genes from transcriptome analysis by RT-qPCR. Values for relative mRNA expression from RT-qPCR are depicted on the left *y* axis. Values for gene expression levels detected by transcriptome analysis are depicted on the right *y* axis as log_10_(FPKM + 1). Blue indicates jejunum, and red indicates ileum. Symbols and letters indicate significant differences obtained by RT-qPCR as follows: a, b, and c indicate significant differences between different time points in the jejunum; A, B, and C indicate significant differences between different time points in the ileum; and # indicates a significant difference obtained between jejunum and ileum at each time point. Error bars show standard errors.

Although *CD79B* was identified as a regionally and temporally DE gene by transcriptome profiling, no significant regional or temporal changes were observed through real-time quantitative PCR (RT-qPCR) (Fig. 9). In addition, the transcriptome analysis showed that *CD4* and *IL17F* were identified as JE-enriched genes at D7 and D14, respectively, while they were both identified as JE-enriched genes from D7 to D21 by RT-qPCR. The expression of *CLDN2*, *CLDN16*, *MUC5B*, *C8B*, *TLR1LA*, *TLR2t1*, *IL1B*, *IL8*, and *PIGR* by RT-qPCR was consistent with the transcriptome profiling data. In general, for the 12 genes tested, the transcriptome profiling results were mostly confirmed by RT-qPCR analysis, therefore confirming the reliability of our microarray data.

## DISCUSSION

To understand the interaction between the intestinal mucosal immune system development and commensal microbiota colonization, we initially focused on studying the bacterial colonization process in the small intestine of pigeon squabs. Alpha diversity results suggested that ileal bacterial richness and diversity were altered significantly from D0 to D7 but were relatively stable between D7 and D21. Similarly, microbiota colonization occurs immediately after the birth of humans and most animals with the ingestion of varied microorganisms from the environment and their parents (28). Phylogenetic beta diversity analysis revealed that the ileal microorganism composition at birth was different from the three other time points. Taken together, our results implied that the first week after hatching seemed to be the critical window for microbiota colonization in the ileum of pigeon squabs. Despite small-scale changes in the richness and diversity of microbiota during the later stages of development, clear changes were observed in microbial abundances at both the phylum and genus levels as the birds aged. These results indicated that the gradual succession of the intestinal flora continued toward the end of 21 days of age. In other words, a potentially stable microbiota had not yet been well established at 21 days of age. The decreased diversity during the first week after hatching seems to be logical because the newly hatched pigeon squab encounters varied microorganisms from pigeon milk and environment that are not true colonizers but only transient organisms, as further evidenced by the quick subsidence of the majority of this initial microbiota as the true colonizers start to take hold.

With increasing age, the representatives of Proteobacteria were gradually succeeded and replaced by the representatives of Firmicutes in the present study, which coincides with the dynamic changes in microbiota of precocial birds (29). Many members of Firmicutes are related to digestion and fermentation involved in the process of metabolism of starch and play a certain role in energy production (29). It is well known that pigeon squabs are fed only pigeon milk, consisting primarily of proteins and lipids, within the first week of life; as squabs grow, pigeon milk is gradually mixed with grains from the parents’ diets and ultimately replaced by a complete grain diet (30). Thus, the increased abundance of Firmicutes with age is expected, as pigeon squabs start to ingest carbohydrates. A recent study showed an abundant and diverse microbiota in pigeon milk, dominated by representatives of the phyla Firmicutes that can be transmitted to squabs (31). These findings may imply that the microbiota in pigeon milk also participate in the intestinal bacterial colonization process of newly hatched squabs. Many representatives of the phyla Proteobacteria, such as Escherichia coli and Salmonella, are considered pathogens in humans and animals, with well-established proinflammatory mechanisms (29). It has been proposed that an elevated imbalance in the intestinal microorganisms, especially for Proteobacteria, can be a sign of disease in the host (32); therefore, the decreased abundance of Proteobacteria as the birds aged in this study may potentially imply that pigeon squabs were undergoing healthy development. Within the first week of microbiota colonization, Escherichia gradually increased, becoming predominant in the small intestine of pigeon squabs at D7. This phenomenon is consistent with the findings of Schokker et al., who found that Escherichia was dominant in the early life of broilers (17). Although the role of intestinal commensal Escherichia is not yet fully understood, we need to acknowledge its dominance in early life and potential to play a certain role in developing the intestinal microbial ecosystems in pigeon squabs. After D7, Escherichia was outcompeted by representatives of Firmicutes and Actinobacteria, and by the age of 21 days, Lactobacillus became the predominant genus in the small intestine of pigeon squabs, exceeding 90% of the relative abundance. Consistent results were obtained in a study of chickens, where it was shown that Lactobacillus comprised approximately 90% of the microbiota of the small intestine (33). Lactobacillus is beneficial for health due to its role in producing lactic acid, which can inhibit pathogen growth. Some Lactobacillus strains have been shown to alleviate diarrhea in mammals by modulating the microbiota community and strengthening immune system function in the small intestine (34). This increased relative abundance of Lactobacillus with age may imply its likely beneficial role in regulating the intestinal health of pigeon squabs.

The jejunum and ileum have been considered the crucial site of intestinal interaction between the host mucosa, ingested food, and microbiota (35, 36). In the present study, we observed a remarkable difference in the regional and temporal expression patterns in the small intestine of pigeon squabs by transcriptome analysis. Regionally, DE genes were mainly enriched in biological functions corresponding to the development process, transport, and the metabolic process, which might reflect the major differences in morphological and functional developments between the jejunum and ileum in the maturation of pigeon squabs. This result coincides with what we know about the intestinal development of pigeon squabs provided by our previous work, which used other traditional methods, such as enzymatic activity analysis and histological observations (30). In contrast, temporal gene expression patterns were similar between the jejunum and ileum, and the differences between the two intestinal segments were mainly determined by expression levels. The genes belonging to clusters with high expression immediately after hatching and decreasing expression later displayed several features of metabolic and transport functions according to the functional annotation clustering analysis. In contrast, genes belonging to clusters with low expression at hatching and increasing expression later encoded features corresponding to immune functions. These temporal expression patterns are consistent with previous observations in mammals and precocial birds (3, 37).

Because we aimed to study the development of the intestinal immune system, further analyses of the expressed immune-related genes were performed. According to the hierarchical cluster analysis of these genes, a clear separation of their expression profiles between the jejunum and ileum was also observed, which coincides with what we observed from the whole-transcriptome profiling. This result suggests that the mucosal immune system in the jejunum and ileum might be undergoing markedly different development during early life in pigeon squabs. KEGG analysis of regionally DE immune-related genes revealed enriched pathways, such as cytokine-cytokine receptor interaction and cell adhesin molecules, which might suggest a significant difference between the two intestinal segments in relation to immune responses at both the innate and adaptive levels. The expression profile of immune-related genes in the jejunum and ileum at the day of hatching differed from that in the other three age groups, indicating that the development of the immune system of the small intestine is very dynamic during the first week posthatch in pigeon squabs. This result seems logical, because after hatching, dietary antigen and bacterial colonization trigger intestinal immune maturation of Aves (3). Analysis of temporally DE immune-related genes based on KEGG pathway enrichment showed that 6 enriched pathways, including cytokine-cytokine receptor interaction, the Toll-like receptor signaling pathway, and the intestinal immune network for IgA production, overlapped in the jejunum and ileum, indicating that these pathways might play crucial roles in intestinal immune system development at one or more of the time points. However, future *in vitro* or *in vivo* target validation experiments are necessary to provide a more convincing interpretation of the roles of these pathways in modulating the intestinal immune system development.

To further understand the development of the mucosal immune system in the small intestine of pigeon squabs, specific innate and adaptive immune-related genes were subjected to further analysis. There were significant differences in the expression of these genes between the jejunal and ileal regions. For instance, higher expression levels of genes encoding TJ proteins, mucins, antimicrobial peptides, TLRs, T- and B-cell markers, and cytokines and higher concentrations of SIgA were detected in the jejunum than in the ileum, whereas higher expression levels of complement genes were detected in the ileum than in the jejunum. The results obtained were not in complete agreement with those previously presented in the literature. For instance, in newborn calves, the expression of complement, TJ protein, and IgA complex genes was upregulated while the expression of TLR, B-cell marker, antimicrobial peptide, and cytokine genes was downregulated when comparing the jejunum to the ileum (37). However, studies on the regional transcriptome differences in the small intestine of altricial birds, as well as precocial birds, appear to be scarce, and we have not been able to find any other published data with which to compare with our results. Regardless, these regional differences of the specific innate and adaptive immune-related genes in the present study indicate that the jejunum rather than the ileum plays a crucial role in immunity at both the innate and adaptive levels in pigeon squabs during the early posthatch period. Our observations may help to explain the phenomenon of the occurrence of intestinal infections mainly in the ileum of pigeon squabs.

In addition, a remarkable temporal difference in the innate immune-related genes was detected in this study. Genes encoding TJ protein families, such as *CLDN2*, *CLDN15*, and *CLDN16*, were upregulated in the jejunum within the first week but showed no temporal change in the ileum. These claudins have been proven to increase cation-selective paracellular permeability and, therefore, can be considered pore-forming claudins (38). In contrast, increased expression of pore-sealing claudin genes, such as *CLDN1* and *CLDN4*, was detected both in the jejunum and ileum 1 week postpartum in mammals (37). However, our observations are comparable to findings in precocial birds, where intestinal *CLDN2* expression is increased immediately after hatching (39). From the literature, it is known that *CLDN2* and *CLDN15* enhance the proliferation of normal cryptic cells (39, 40). We propose that the increased expression of *CLDN2* and *CLDN15* during posthatch development probably reflects the formation of crypts. Consistent with this interpretation, our previous work has revealed that crypt depth increases nearly 3-fold in the jejunum within the first week posthatch of pigeon squabs; however, changes in the ileum were smaller than in the jejunum (30). Because *CLDN16* serves as a calcium ion channel subunit in the paracellular pathway and is detected in large amounts in mature goblet cells, it plays a crucial role in calcium ion-related mucus secretion by goblet cells (41). Thus, it is probably not surprising to find a temporal increase in *CLDN16* expression accompanied by a temporal increase in *MUC5B* expression in this study. Within the first week after hatching, increased expression of genes encoding cytokines (*IL1B* and *IL8*) and PRRs (*TLR1*, *TLR2*, *TLR7*, and *NLRC4*) and decreased expression of genes encoding complements (*C4* and *C8B*) and antimicrobial peptides (*CAMP*) were also observed in both the jejunum and ileum. *MUC5B* is a major component of the secreted mucins in the protective mucus layer that resists the harsh luminal environment (42). *IL1B* and *IL8* are mainly produced by innate immune cells and participate in proinflammatory immune responses following the colonization of commensal bacteria (2). PRRs play a pivotal role in recognizing invasive pathogens, as well as commensal microorganisms, and initiating inflammatory and immune responses (43). One important function of the complement system in the immune system is the role it plays in the inflammatory response to microbes (44). *CAMP* produced by neutrophils is a member of the antimicrobial peptide family that is present in humans and animals and has potent bactericidal and immunoregulatory activities (19). Accordingly, we expected increases in the expression of these innate immune-related genes in the intestine concomitant with microbiota colonization. To our surprise, the expression of complement and antimicrobial peptide genes was downregulated during the first week after hatching. Although the exact mechanism for this decrease has not been explored, due to the decreased expression of complement and antimicrobial peptide genes, as well as the increased expression of pore-forming claudins, the penetration of bacteria across the mucosa may be strengthened within the first week of life. Translocated bacteria may very well result in upregulated gene expression of *IL1B* and *IL8*. As expected, the expression of *IL1B* and *IL8* followed the same kinetics as the expression of *TLR1*, *TLR2*, *TLR7*, and *NLRC4* in the current study, indicating that these PRRs have potentially important roles in the inflammatory response to the colonization of commensal bacteria. This finding is also supported by the enriched Toll-like receptor signaling pathway of temporally DE immune-related genes and by the strong correlation observed between the relative abundances of bacterial taxa and the mRNA expression of PRRs during the first week of life. In particular, temporal changes of the genera Escherichia and Lactobacillus were also positively correlated with those of *IL1B* and *IL8* in the ileum during the same development period. It seems that colonization by Escherichia and Lactobacillus within the first week of life is likely one of the causative factors for induction of the expression of *IL1B* and *IL8* in the ileum of pigeon squabs. Because this study is the first attempt to explore microorganisms correlated with immunoregulation in pigeons, further studies are needed to verify whether the microorganisms identified in the current study as correlated with cytokine signal transduction really have immunoregulatory activities. In the ileum, the majority of the above-mentioned innate immune-related gene expression, as well as the richness and diversity of the microbiota, reached stable levels after D7 posthatch. Therefore, we assumed that ileal innate mucosal immune system development regulated by microbial ecosystems occurs mainly in the first week and that functional maturation is attained in the second week of life or even later.

A remarkable temporal difference in the adaptive immune-related genes was also detected in the current study. All temporally DE T- and B-cell lineage-specific genes showed markedly increased expression through the 3 weeks after hatching in both the jejunum and ileum. This temporal pattern was similar to that of chicks. In chicks, immature T and B cells are already detected in gut-associated lymphoid tissue at the time of hatching, and functional maturation occurs during the first 2 weeks after hatching (27). In addition to the noted changes in T-cell lineage-specific genes, cytokine gene (*IL16*, -*17F*, *-22*, and *-26*) expression was upregulated within the first week. *IL16*, a chemoattractant, plays a major role in regulating the activation, proliferation, and recruitment of T cells and promoting the generation of proinflammatory cytokines (45). *IL17F*, *IL22*, and *IL26* are potent proinflammatory cytokines secreted by the Th17 subset and are crucial in regulating mucosal antimicrobial immune responses (46, 47). However, we did not find any temporal differences in the production of Th1, Th2, or Treg cytokines, especially anti-inflammatory cytokines. Inconsistent with our observations, the elevation of both proinflammatory and anti-inflammatory cytokines was observed in mammals and precocial birds during postnatal development (24, 37, 48). In mammals, the production of IL-1β induced by commensal bacteria is a key step for the differentiation of the Th17 subset in the intestine (49). Because the expression of Th17 cytokines followed the same kinetics as *IL1B* within the first week, the upregulation of Th17 cytokines in pigeon squabs is also likely to be induced by IL-1β, which is produced in response to the colonization of commensal bacterial in the ileum, as we mentioned above. The temporal expression pattern of proinflammatory cytokines in the present study might imply that cellular adaptive immune responses mediated by Th17 cells following commensal-induced IL-1β production in the ileum begin as early as the first week posthatch, but this cellular immunity seemed to be less effective in terms of maintaining the inflammatory response balance. However, this imbalance period may be considered necessary for adequate immune development and homeostasis establishment in the small intestine, as described in previous studies on Aves (20).

SIgA can help immune tolerance by entrapping invasive pathogens and commensal bacteria when patrolling the mucus and preventing the microbial translocation through the mucosal barrier (23). In the present study, the SIgA concentration in the jejunum remained at a low level for 2 weeks after hatching and increased at week 3. In the ileum, the SIgA concentration remained at a low level through the 3 weeks after hatching. These observations seemed to be logical because the low SIgA levels enabled the colonization of commensal bacterial in the small intestine immediately after hatching. The temporal pattern of SIgA expression obtained in this study is comparable with previous studies in chickens (7). Additionally, in mammals, only scarce expression of SIgA could be detected in the first weeks after birth (50). Moreover, our observation that proinflammatory cytokines are elevated when SIgA is lacking coincides with the proposed functions of SIgA. Therefore, we assumed that there could be less humoral adaptive immunity activity during at least the first 2 weeks of life. Mucosal SIgA is induced in both T-cell-dependent and T-cell-independent pathways in mammals. It has been demonstrated that Th2/Treg cytokines, such as IL-4, IL-10, and transforming growth factor beta, enable mucosal B cells to skew their isotype toward IgA (51). However, in the present study, no correlation was found between IgA and Th2/Treg cytokine gene expression levels. Since each round of SIgA transport consumes one PIGR molecule, the regulation of *PIGR* expression and its epithelial transport is essential to modulate SIgA concentrations in most animals’ external secretions (52). Our transcriptomic analysis revealed that the expression of *PIGR* preceded the production of SIgA. From the literature, it is known that *PIGR* gene expression is probably induced by the proinflammatory cytokine IL-1β and/or Th1/Th2/Th17 cytokines, including those encoded by *IFNG*, *TNFA*, and *IL4*, in mammals and birds (24). In the present study, we found that only the expression of *IL1B* followed the same kinetics as *PIGR*. Collectively, our results seem to imply that mucosal SIgA production in pigeon squabs is T cell independent. Because knowledge about the mechanisms behind class switching to IgA in Aves is scarce, more in-depth research is expected to clarify whether pigeon squab SIgA is induced in a T-cell-dependent or T-cell-independent manner or both.

In conclusion, this is the first study to characterize the succession of microbiota and the spatiotemporal development of the mucosal immune system in the small intestine of an altricial bird during the early posthatch period. As in mammals and precocial birds, there is a mutual relationship between the intestinal mucosal immune system development and commensal microbiota colonization in pigeon squabs, according to our observations on the relationship between the succession of microbiota and the expression patterns of immune-related genes. Our results imply that the first week after hatching seems to be the critical window for microbiota colonization and that a potentially stable microbiota has not yet been well established at 21 days of age. Regional transcriptome differences revealed that the jejunum rather than the ileum plays a crucial role in immunity at both the innate and adaptive levels. In the ileum, temporal deviation in innate immune-related genes mainly occurred in the first week of life and was accompanied by a temporal change in microbiota composition, indicating that intestinal innate mucosal immune system development regulated by microbial ecosystems occurs mainly in this period. Given the role of Th17 in immune homeostasis of animals and humans, our observations of Th17 cell activation as expressed by the synergetic expression of commensal-induced cytokines and Th17 cytokines indicate that cellular adaptive immune responses mediated by Th17 cells following microbiota colonization in the ileum begin as early as the first week posthatch; however, this cellular immunity seems to be less effective in terms of maintaining the inflammatory response balance. Because the induction of high levels of mucosal SIgA seems to take approximately 3 weeks, we favor the idea that humoral adaptive immunity might be less active during at least the first 2 weeks of life. Collectively, our data may help to explain the phenomenon of the occurrence of intestinal infections mainly in the ileum of pigeon squabs during the early posthatch period.

However, there are two limitations to the present study that must be taken into consideration. First, the host-microbiota interactions obtained in this study were based on the potential link between mucosal immune system development and microbiota composition in the ileum; however, future studies are needed to show whether the host-microbiota interactions are segment specific in the small intestine of pigeon squabs. Second, it is important to point out that pigeon parents transfer not only nutrients but also microorganisms to their squabs through the pigeon milk to help them adapt to the dynamic living environment. Thus, it will be necessary to further investigate whether and how the pigeon milk microbiota are involved in the intestinal bacterial colonization process and immune system development of newly hatched squabs. Nevertheless, we provide evidence that colonization by Escherichia and Lactobacillus within the first week of life is likely one of the causative factors for the induction of *IL1B* and *IL8* expression in the ileum. Although we do not elucidate the mechanisms behind the interplay between the commensal bacteria we identified and the proinflammatory cytokine signal transduction, our results are probably useful for researchers as a first step to screen microbes with desirable and/or undesirable immunoregulation characteristics from complex microbial communities.

## MATERIALS AND METHODS

### Ethics approval.

All experimental protocols involving animals were approved by the Animal Care and Welfare Committee of Animal Science College and the Scientific Ethical Committee of Zhejiang University (Hangzhou, China).

### Birds and sample collection.

Pigeon squabs (Columba livia) were obtained from a commercial pigeon farm (Baixiang Pigeon Breeding Co., Ltd., Fuyang, China) and were fed pigeon milk by parent pigeons in a beak-to-beak manner. Twelve birds with similar body weights were selected for sampling at four time points, D0 (day of hatch but before feeding), D7 (day 7 posthatch), D14 (day 14 posthatch), and D21 (day 21 posthatch), for a total of 48 birds. The entire small intestine was removed from each of the pigeon squabs immediately after cervical dislocation. Intestinal tissue samples for transcriptome analysis were obtained from the midpoints of the jejunum and ileum. Sterile phosphate-buffered saline (pH 7.0) was used to remove ingesta from all tissue samples except D0 samples. Because the pigeon species is characterized by rudimentary, nonfunctional ceca (53), samples for microbiota analysis were obtained from the intestinal contents at the distal end of the ileum. All samples were collected in sterile containers, packed carefully, frozen by immersion in liquid nitrogen, and stored at −80°C. Additionally, samples from 12 birds within the same age group were pooled in 4 pools of samples from 3 birds and homogenized to avoid intraindividual variability before further analysis; thereby, each age group had 4 experimental units (pools) for statistical analyses.

### DNA extraction, 16S rRNA gene amplification, and sequencing.

Total bacterial DNA was isolated from the ileal content samples with an E.Z.N.A. stool DNA kit (Omega Bio-Tek, Inc., Norcross, GA, USA), and the V3-plus-V4 region of the 16S rRNA gene was amplified using the following primers: 338F, ACTCCTACGGGAGGCAGCAG-3′, and 806R, GGACTACHVGGGTWTCTAAT. The PCR product was excised from a 2% agarose gel and purified using an AMPure XT bead kit (Beckman Coulter Genomics, Danvers, MA, USA). Sequencing libraries were generated using a TruSeq DNA PCR-free sample preparation kit (Illumina) following the manufacturer’s instructions. Library quantification was performed using a Qubit 2.0 fluorometer (Invitrogen, Carlsbad, CA). Microbiota analysis was performed using the resulting libraries on an Illumina MiSeq system to generate 300-bp paired-end reads. Paired-end reads were joined by using FLASH and filtered by using Vsearch (54, 55). Clean reads were obtained for subsequent analyses after data filtration and chimera removal.

### Microbiome analysis.

Sequencing data were subjected to various microbiota analyses using Quantitative Insights Into Microbial Ecology (QIIME) 1.9.0 and the MicrobiomeAnalyst platform. The analyses included the extraction of operational taxonomic units (OTUs), clustering analysis, and alpha and beta diversity analysis (56, 57). OTUs were clustered using the Vsearch algorithm with a cutoff value of 97% similarity. Alpha diversity, such as the Chao1 richness estimate and Shannon diversity index, was calculated according to the relative abundances of OTUs. Rarefaction curves were constructed by using the Chao1 estimator of species richness with 20 sampling repetitions at each sampling depth. Phylogenetic beta diversity was determined by permutational multivariate analysis of variance (MANOVA) of Bray-Curtis distance metrics according to the relative abundances of OTUs. Nonmetric multidimensional scaling (NMDS) was performed on the resulting distance matrices to generate 2-dimensional plots. Hierarchical clustering based on the Bray-Curtis distance and the Ward clustering method was also performed using the relative abundances of OTUs. Taxonomy was assigned using the Ribosomal Database Project with a bootstrap confidence threshold of 80%. Linear discriminant analysis coupled with effect size (LEfSe) determinations were carried out with an online LEfSe tool to identify the bacterial taxa differentially represented between age groups at different taxonomic levels (58).

### RNA isolation and RNA-Seq library preparation.

TRIzol reagent (Invitrogen) was used to isolate total RNA from jejunal and ileal tissue samples. The quality of the extracted total RNA was evaluated by native RNA electrophoresis on a 1.0% agarose gel, with the UV absorbance ratio at 260 and 280 nm, using the 2100 RNA 6000 nano assay kit (Agilent Technologies, CA, USA). Total RNA samples with an integrity number greater than 7.0 were used for construction of cDNA libraries with the TruSeq stranded total RNA sample preparation kit (Illumina, San Diego, CA), following the manufacturer’s directions. Library quantification was performed using a Qubit 2.0 fluorometer (Invitrogen, Carlsbad, CA). The resulting libraries were subjected to transcriptome analysis (RNA-Seq) to produce 100-bp paired-end reads on an Illumina HiSeq 2000 system (Illumina). All reads were demultiplexed based on their index sequences with CASAVA version 1.8 (Illumina), and reads that did not pass the Illumina chastity filter were discarded.

### Gene expression level analysis.

The filtered reads were aligned onto the reference genome (Cliv_1.0) using HISAT version 2.0 with default parameters (59). To count the read numbers mapped into each gene, HTSeq version 0.6.1 was used with the parameter -m union (60). The expression levels of mRNAs in each sample were estimated as fragments per kilobase of transcript per million mapped reads (FPKM). We called a gene “expressed” in a given sample if it had an FPKM value of ≥0.1 according to the distribution of FPKM gene expression levels. Commonly expressed genes overlapping the intestinal segments were identified using Venn diagrams. To visualize the differences in the expressed genes among all the samples, principal-component analysis (PCA) was performed on scaled variables using the R statistics function prcomp(data), and graphics were created with the ggplot2 R package (3.3.2).

Regionally differentially expressed (DE) gene analysis was carried out with the DESeq R package (1.18.0), and a fold change of ≥2 and false discovery rate (FDR) of <0.05 were used as the criteria to screen DE genes. Temporally DE gene analysis was carried out with the maSigPro R package (1.60.0). First, the least-squares technique was used to adjust this global model for identifying DE genes, and significant genes were selected by setting the FDR at 0.05 with a minimum observation of 20. Second, the “backward” step method with “alfa” at 0.05 was used to generate a stepwise regression fit for time series gene expression experiments. Then, lists of DE genes according to each variable were generated based on the *r*^2^ value (>0.8). After maSigPro analysis, a fold change of >2 was also required to consider a gene DE.

### Enrichment analysis.

DE genes were enriched for Gene Ontology (GO) biological processes and Kyoto Encyclopedia of Genes and Genomes (KEGG) pathways with a Cytoscape plugin, known as ClueGO (61). In this enrichment analysis, the Columba livia GO and KEGG databases were used as the background. Each analysis was carried out with the medium network type option. A two-sided hypergeometric test with an FDR of <0.05 was used to select significant GO terms/pathways. The functional annotation clustering option was used for GO enrichment analysis. The functional network was visualized using a prefuse force-directed-layout algorithm. The maximum interval level of the GO tree was 8, and the minimum level was 2.

### Analyses of specific genes related to the mucosal immune system.

We obtained the mucosal immune-related gene list from the InnateDB database and ImmPort database (62, 63). Immune-related genes expressed in both the jejunum and ileum were identified using Venn diagrams. To compare the expressed immune-related genes of the individual samples, hierarchical clustering among the samples was carried out according to the Euclidean distance and the Ward clustering method, and the results were displayed as a dendrogram plot using the factoextra R package (1.0.7).

Genes encoding TJ protein families, mucins, antimicrobial peptides, and complements were collected to explore the expression patterns of innate immune-related genes. Genes encoding TLR and NLR proteins were collected to explore the expression patterns of innate immune sensing-related genes. Genes encoding subunits of cluster of differentiation (CD) proteins were collected to explore the T- and B-cell lineage-specific markers. A cytokine list was obtained from the KEGG pathway annotation “Cytokine-cytokine receptor interaction” as described by Liang et al. (37). PCA biplots of those selected genes were prepared using ggbiplot(prcompt) as part of the ggplot2 library package in the R platform.

### Determination of intestinal mucosal SIgA concentrations.

Jejunal and ileal tissue samples were homogenized on ice in 10 volumes of normal saline and centrifuged at 4°C for 20 min at 20,000 × *g*. Then, the resulting supernatant was collected for further measurements. SIgA concentrations were examined via a sandwich enzyme-linked immunosorbent assay (ELISA)-specific SIgA quantitation kit (Shanghai Mlbio Institute, Shanghai, China) on a SpectraMax M5 microplate reader (Molecular Devices, Sunnyvale, CA), following the manufacturer’s instructions. The protein concentrations in the jejunal and ileal tissue samples were measured via Coomassie brilliant blue G-250 reagent with bovine serum albumin (BSA) as a standard. The SIgA concentrations were estimated as nanograms per gram of protein.

### Validation of mRNA expression by RT-qPCR.

*CLDN2* and *CLDN16* from TJ protein-coding genes, *MUC5B* from mucin protein-coding genes, *C8B* from complement protein-coding genes, *TLR1LA* and *TLR2t1* from TLR-coding genes, *CD79B* as the B-cell marker, *CD4* as the T-cell marker, *IL1B*, *IL8*, and *IL17F* from cytokine protein-coding genes, and polymeric immunoglobulin receptor (*PIGR*) from the genes encoding immunoglobulins were collected to verify their regional and temporal differential expression patterns. cDNA was synthesized by Moloney murine leukemia virus (M-MLV) reverse transcriptase (TaKaRa, Dalian, China) from 2 μg of total RNA extracted as described above, following the manufacturer’s instructions. Gene-specific primers for those 12 genes, as well as the endogenous glyceraldehyde-3-phosphate dehydrogenase reference gene (*GAPDH*), are shown in Text S1. The abundance of mRNA was examined through a SYBR premix PCR kit (TaKaRa) according to the manufacturer’s instructions via a StepOne Plus real-time PCR system (Applied Biosystems, Foster City, CA). The experiments were performed in triplicate for each data point. The difference in the cycle threshold (*C_T_*) value for *GAPDH* was less than 0.5 among all samples, and thus, it was considered to be an appropriate endogenous control. The 2^−ΔΔ^*^CT^* method was adopted to calculate the relative quantification of gene expression (64).

### Statistical analysis.

In the current study, the Benjamini and Hochberg multiple-testing correction was used to calculate FDR. Significant regionally and temporally DE genes were declared at a fold change of >2 and FDR of <0.05. A two-sided hypergeometric test with an FDR of <0.05 was used to select significant GO terms/pathways. The effect of age on microbiota diversity and richness was assessed by one-way analysis of variance (ANOVA) followed by the Kruskal-Wallis test, and a *P* value of <0.05 was selected as the level of significance. The effect of age on microbiota compositions was evaluated by LEfSe analysis with the Kruskal-Wallis test and FDR correction. Bacterial taxa with an FDR of <0.05 and linear discriminant analysis (LDA) score of >2 were selected as biomarker taxa. The SIgA contents and RT-qPCR results were analyzed using the unpaired Student’s *t* test between two intestinal segment groups and one-way ANOVA followed by Tukey’s multiple-comparison test among four age groups, respectively, and were declared significant at a *P* value of <0.05. The correlations between bacterial abundance and gene expression in the ileum were assessed by Pearson correlation coefficients. All genera were compared against each gene expression profile for each time point. The correlation was considered significant at a *P* value of <0.05.

### Data availability.

The data sets presented herein can be found in the NCBI Sequence Read Archive and in the supplemental material. The raw sequencing data have been deposited in the NCBI Sequence Read Archive database (accession number PRJNA811402).
